# Optical fiber-based open source low cost portable spectrometer system

**DOI:** 10.1016/j.ohx.2024.e00530

**Published:** 2024-04-18

**Authors:** Gatis Tunens, Ernests Einbergs, Katrina Laganovska, Aleksejs Zolotarjovs, Karlis Vilks, Linards Skuja, Krisjanis Smits

**Affiliations:** Institute of Solid State Physics, University of Latvia, Kengaraga Str. 8, Riga LV-1063, Latvia

**Keywords:** Spectroscopy, Fibers, Fluorescence, Absorption

## Abstract

This article explores the development of a small, compact fiber-based spectrometer system designed to overcome the limitations of standard spectrometers, such as the high cost and restricted accessibility.

Operated by a Raspberry Pi, the fiber-based spectrometer system uses the increased computing power to provide versatile modes of operation and powerful data processing, while maintaining a small size. Specifically crafted for basic chemistry and biology lab setups, where fibers allow measurements in different conditions, and customization enables fluorescence, light scattering, and absorption measurements.

The system is adaptable and versatile, offering ease of modification and adaptation for a broad range of applications.

## Graphical abstract

**Figure:**
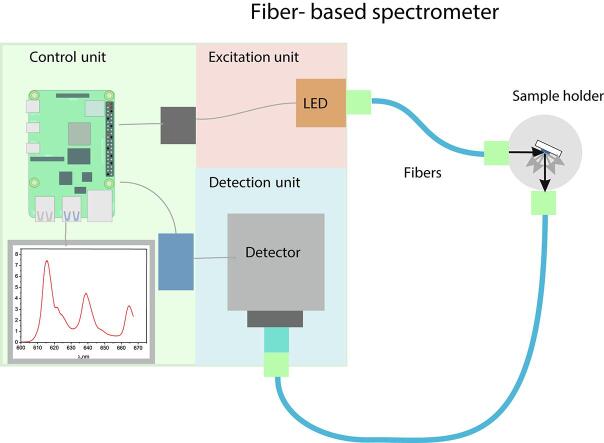

**Specifications table:** 

Hardware name	Optical fiber-based open source portable spectrometer system
Subject area	• Engineering and materials science • Chemistry and biochemistry • Biological sciences (e.g., microbiology and biochemistry) • Environmental, planetary and agricultural sciences
Hardware type	• Measuring physical properties and in-lab sensors
Closest commercial analog	Hardware is based on the Hamamatsu C12880MA spectrometer chip, therefore close commercial comparison could be made with compact CCD spectrometers like the Thorlabs CCS100 (€ 2.110,94), although our presented system supports a broader range of applications.
Open source license	GNU General Public License (GPL) 3.0
Cost of hardware	€ 400 (excluding fibers)
Source file repository	*https://doi.org/10.17605/OSF.IO/VGYN7*

## 1 Hardware in context

Traditional optical spectrometers, with their high costs and large physical footprint, are hardly affordable for workgroups with limited funding and are unsuited to field scientists. Low-cost and compact spectrometers resolve these issues by making various spectroscopy methods accessible to a broader audience, such as students and amateurs [1], [2].

Modern spectrometers often use large monochromators to reach excellent resolution, and iCCD cameras to multiply incident photons, leading to superb sensitivity, and signal-to-noise ratio. However, many applications in various scientific fields do not require high sensitivity and/or spectral resolution [3], [4], [5], [6], [7]. Portable fixed-grating spectrometers are commonly used in these cases. They can have a considerably high resolution and usually cost between 2 and 10 thousand dollars. Although they are currently used in most in-field applications, there are significant possibilities for further miniaturization and cost reduction.

Another important factor to consider is the adaption of equipment to a wide range of applications. Commercial equipment often comes with closed software and a rigid technical solution, which is challenging or, in most cases, impossible to modify. This is troublesome for emerging researchers and impedes the development of new methodologies and innovation [8].

The design of an optical system is the most complicated part of designing a spectrometer-based application. However, this challenge can be effectively addressed by integrating optical fibers. Interest in fiber-based systems has grown over the past few decades. In contrast to their conventional counterparts, fiber-based spectrometer devices stand out for being portable and compact. Their key strengths include simple integration and customisation, user-friendly setup, and plug-and-play functionality. They are thus especially well-suited for consumers that lack the required expertise for effective alignment, calibration, and operation of optical systems. Additionally, the fiber design allows hermetic sealing and the creation of robust systems that are usable in dusty or humid environments [9].

While various projects have explored the creation of portable spectrophotometers for on-the-go use, most of these initiatives prioritize a low-cost device designed for educational purposes or very basic measurements, rather than ensuring the reproducibility, and accuracy required for scientifically significant data acquisition [5], [10], [11], [12], [13], [14], [15].

The Hamamatsu C12880MA spectrometer chip is the most cost-effective commercial spectrometer available, to the best of our knowledge to date. This ultra-compact, low-cost device provides a quick and simple approach to measuring light spectra. It consists of only three components: a fixed entrance slit, a reflective concave diffraction grating, and a CMOS sensor. The reduced number of components, and the substitution of multiple focusing mirrors with a single concave diffraction grating, allow for reduced stray light, and ultra-small physical footprint while maintaining adequate sensitivity [16]. The biggest downside of the miniature spectrometer is the limited spectral resolution.

We previously released an open-source hardware (OSH) spectrophotometer solution that uses the Hamamatsu C12880MA, which has gained popularity and extensive usage. However, our earlier version has several limitations. It is constrained to measurements done in a transmission mode, and its computational capabilities and speed are limited, since it iss Arduino-based [17]. Therefore, to address these limitations, and to expand the applicability of the system, in this article, we propose an easily customizable Raspberry Pi-controlled fiber-based spectrometer system.

## 2 Hardware description

A typical spectroscopy measurement system can be divided into the following components: a light source (I), an optical system (II), a sample holder (III), a detection unit (IV), and a control unit (V).

**Fig. 1:**
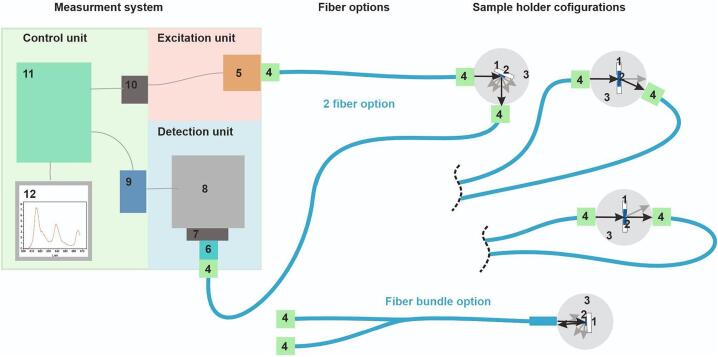
Schematic representation of the measurement system 1-sample holder, 2-sample, 3-sample chamber, 4-SMA905 connector, 5-LED, 6-lens, 7-filter, 8-spectrometer, 9-signal processing circuit, 10-LED power circuit, 1-Raspberry Pi, 12-display.

In the present system, we tried to integrate various units, namely excitation, detection, and control units, within a single mechanical housing referred to as the main unit (Fig. 1). Simultaneously, the utilization of a fiber-based design offers significant flexibility and adaptability of the optical path, allowing for extensive variations and customization options. For instance, alternative external light sources and a variety of sample holders can be employed. In this particular case, we tested 2 LEDs (370 nm, 480 nm) and two optical fiber solutions: fiber bundle (according to Thorlabs: Dip Probe Bundle) and two-fiber systems, although several sample holder configurations are possible. These options enable various types of spectral measurements. For instance, sample holder options A and D can be utilized for luminescence, option C for absorption, and options B and D for scattering measurements (Fig. 1).

In principle, these kinds of fiber-based systems have a wide range of uses; the control and sensing units serve as the foundation of the system, with everything else being adjustable in a variety of ways. For particular applications, we strongly advocate using costlier lasers and/or optical components.

### 2.1 Main unit

**Fig. 2:**
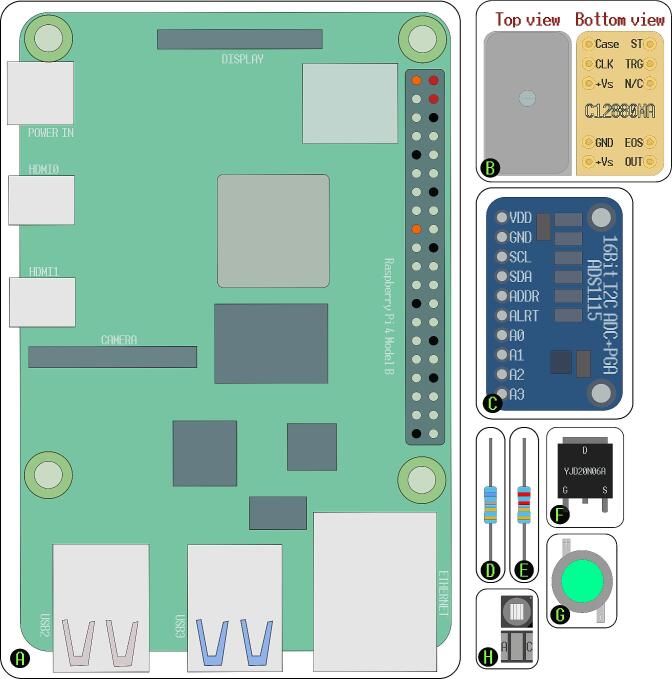
Visual representation of electronic components. (A) Raspberry Pi 4B; (B) Hamamatsu C12880MA; (C) ADS1115 Analogue/Digital converter (A/D converter); (D) 6.8 Ohm resistor; (E) 2.2 Ohm resistor; (F) YJD20N06A MOSFET; (G) 480 nm LED; (H) 370 nm LED.

The first part of our system description will focus on the primary unit (measurement system), which includes the components that manage excitation, signal detection, and control. All electronic components (Fig. 2) are placed in the main unit, forming one electrical circuit.

The following components were used:

• Raspberry Pi 4B;
• Hamamatsu C12880MA Mini-spectrometer;
• ADS1115 Analogue/Digital converter (A/D converter);
• YJD20N06A enhancement type n-channel MOSFET;
• 6.8, 2.2 Ohm resistors, at least 0.5 W rated;
• 480 nm LED, 3.2–3.6 V, up to 700 mA (generic, no serial number);
• 370 nm LED, 3.3–3.8 V, up to 500 mA (PK2B-2LLE-VS).

The components are easily replaceable by alternatives, although they should retain compatibility with the open-source software provided in this article (instructions will be added within the software comments if minor software modifications are required). The only irreplaceable component is the Hamamatsu C12880MA spectrometer since it has preset timing rules that have to be followed for proper functionality. The supply voltage for Hamamatsu C12880MA must be 5 V, and it outputs an analog 5 V signal. The device is interfaced with 2 input pins that recognize logical signals within the 3 to 5 V range.

The Raspberry Pi 4B is a widely used microcontroller system with enough computational power to be used as a personal computer. It has a 40-pin General purpose input and output (GPIO) array that natively supports a wide range of popular communication protocols (i.e. UART, SPI, I2C). Using a method called bit-banging, any of the unused GPIO pins can be configured to run these or other 3 V3 protocols, although custom software is required, and these approaches may lead to slower communication times due to being CPU-bound. It should be noted that the GPIO pins can also be used as 5 V logic level outputs as 3.3 V is high enough to trigger 5 V inputs, but the GPIO cannot be run as inputs at voltages above 3.3 V. The device comes with 4 USB-A connections, 1 Ethernet port, 2 HDMI interfaces, one DSI, and one CSI interface. A 5 V and 3A USB-C type power source has to be used. The Raspberry Pi 4b can be replaced with any other compatible microcontroller, that is capable of running Python code, supports 3 V3/5V logic level output, 5 V power delivery, has the communication interfaces necessary for the analogue to digital converter (ADC), and has enough power to run all of the connected components. The 4B model was chosen only due to its popularity, making it a highly accessible platform. If a system other than the specified Raspberry Pi is utilized, modest changes to the pin definitions and interactions will be necessary.

An ADC is mandatory to register the signal from Hamamatsu C12880MA. A 16-bit ADS1115 ADC was chosen [18] with a generic breakout board. The chip uses I2C protocol to communicate [19] and has a built-in programmable gain amplifier (PGA) with a voltage reference. A premade open-source Python library created for the ADS1x15 series chips provided by Adafruit was used to communicate with the chip. 16-bit precision was deemed necessary for low signal (e.g., fluorescence) measurements after preliminary tests with 8 and 12-bit devices showed subpar results. Any conventional stand-alone ADC chip supporting 5 V analog inputs could be used as a replacement in this setup.

The YJD20N06A n-channel enhancement type MOSFET was used as a generic LED power delivery control switch within this setup, so any compatible transistor with the remainder of the components can be used instead. Note that when choosing a different transistor, consider its maximum gate threshold voltage, as it has to be within the range of the output voltage of your microcontroller − in the Raspberry Pi 4B case, the GPIO pins output a 3.3 V signal when pulled HIGH. If no light source power switching and no pulse width modulation are required, the transistor can be removed from the system entirely, and a direct connection to power can be used if the light source is compatible and properly protected.

Two different LEDs are used with different wavelength output maximums −480 nm and 370 nm. The LEDs can be replaced by other power source-compatible LEDs with peak maximum wavelengths suiting the needs of the intended experiment.

### 2.2 Fibers

An efficient collection of scattered light from the probed sample depends on various optical components involved in the sampling process. The key characteristics of fiber-optic probes include the numerical aperture (NA) and the cross-sectional area (S) of the light-guiding core of the fiber. In this particular case, the system is planned for use in luminescence studies, so in most cases, ultraviolet (UV) light for excitation will be used. The optical fiber must have good UV transmission and low UV-induced absorption (“solarization”). Fibers of this kind have pure silica core, a lower refraction index cladding made of fluorine-doped silica, and hydrogen doping to protect against solarization. Typically, commercially available optical fibers of this type have an NA of 0.22, resulting in a 25° full light acceptance angle. For optimal light collection into a spectrometer, the fiber core diameter should be equal to or very slightly larger than the height of the Hamamatsu spectrometer input slit (0.5 mm). For excitation fiber, a larger diameter core will provide more light, unless a laser source is used.

We used fibers manufactured by CeramOptec that were available in our laboratory. Other choices can be found in optics catalogs, such as the alternative products provided by Thorlabs.

### 2.3 Excitation sources

LEDs and UV Laser Diodes (LDs) are now widely accessible at affordable prices due to their extensive use in light illumination. LEDs are cost-effective and easy to use, emitting non-coherent light with a wide range of wavelengths. On the other hand, LDs generate light that is coherent and focused, but they require precise temperature regulation and a more advanced power source.

Moving on to the laser or LED-to-fiber coupling, various solutions exist, ranging from basic mechanical setups to advanced fiber launch solutions with higher costs. Coupling efficiency primarily depends on the size and intensity angular distribution of the light source, the core diameter, and the numerical aperture (NA) of the connected fiber. Small-diameter or highly collimated (laser) light sources, large core diameters, and high NA values contribute to reduced insertion losses. If the light source is so large and close that it covers the entire acceptance angle of the fiber (∼25°), the simple butt-coupling provides the maximum possible collection efficiency, and no additional optical elements can improve it. In other cases, additional focusing lenses can greatly increase the insertion efficiency, which for laser sources can approach 100 %.

For demanding measurements and if the budget allows we recommend using external lasers or fiber-based lasers.

In traditional luminescence setups, filters are used, which block the excitation light, scattered by the sample, from entering the spectrometer and transmit the longer-wavelength luminescence light. This helps to detect the typically much weaker luminescence emission. By selectively removing excitation light, the signal-to-noise ratio is significantly improved, and background interference is decreased.

### 2.4 Enclosure for the main unit

3D models were created with Autodesk Fusion 360 software and sliced with Ultimaker Cura 4.12.1. Parts were 3D printed using Creality CR-10S Pro with a 0.4 mm nozzle installed. All models utilized a printing speed of 80.0 mm/s and a nozzle temperature of 235 ℃. Part designs are optimized for fused filament fabrication while being mindful of structural integrity; black PETG material was used. Parts are designed to facilitate support-less fabrication.

We offer 2 versions of the main enclosure units. The first iteration is fully 3D printed and uses a slightly cheaper C12880MA spectrometer unit without built-in SMA support (as in C12880MA-20). Although the provided version is fully functioning, the sensitivity of the system is relatively low and might be not suitable for low-light conditions. The second iteration is partially 3D printable. It is possible to print all of the components, but it is advised to machine the lens tubes with a lathe (a step file is provided), as during our testing of a fully 3D printed system, the 3D printed tube did not provide good enough precision and reproducibility to achieve correct lens positioning for good light focus on the optical fiber ends, resulting in a significant loss of signal. Additionally, fully modifying the optical system to include SMA connectors in every stage of the light path (i.e. fiber-coupled laser/LED) would boost the signal sensitivity even more (which would be useful in applications where the expected signal is weak), although increasing the total system cost.

#### 2.4.1 First iteration

**Fig. 3:**
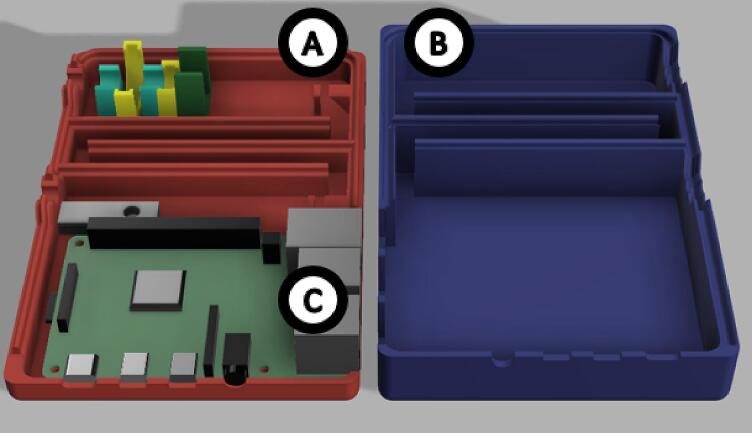
Digital rendering of the whole instrument housing unit. A) Socket unit (BoxBase), B) lid of the housing unit (BoxLid), C) location of Raspberry Pi 4 model B.

The dimensions of the instrument housing unit are 98.0 x 145.0 x 38.0 mm (Fig. 3). It consists of 2 parts. The base and the lid should be printed with at least 50 % infill with a layer height of 0.2 mm or less, which would take approx. 4 h 30 min and 69 g (23.03 m) of filament to print the base and 6 h 14 min and 100 g (33.49 m) to print the lid. Optical configuration is integrated into the base of the instrument housing unit (Fig. 3, A). It is highly advisable to reduce the print speed to 50 mm/s to achieve a higher quality final product. Reduction in speed would result in an approx. print time of 6 h and 33 min for the base and 9 h 7 min for the lid.

The optical configuration for the spectrometer consists of 2 lenses. The first lens quasi-collimated the light exiting the fiber and the second lens focused the light on the spectrometer input slit. In total 3 lenses are required, 2 for the sample chamber and 1 for focusing light from the LED into a fiber.

#### 2.4.2 Sample chambers used with the first iteration design

**Fig. 4:**
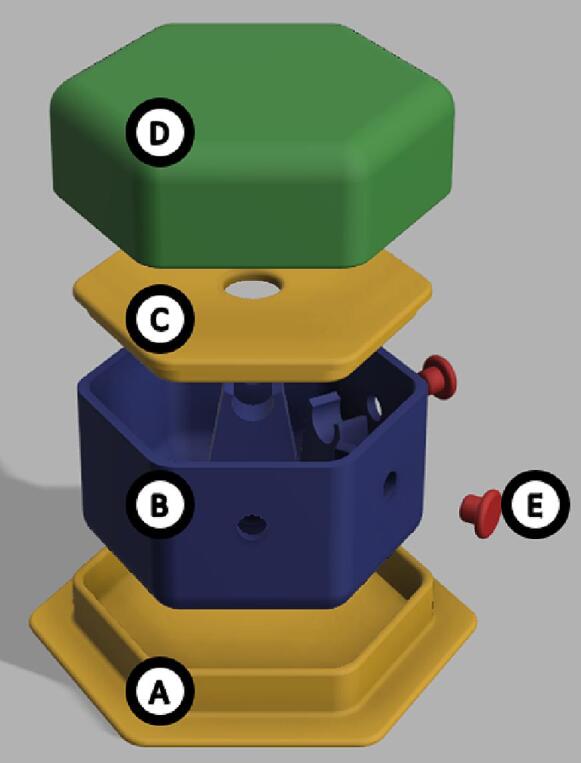
Digital rendering of the sample chamber used with the first iteration design. A) Optional base for added stability (*Measurement Chamber v2 Base*), B) the main sample chamber (*Measurement Chamber v2*), C) lid of the sample chamber to center the micro-centrifuge tube (*Measurement Chamber v2 Epindorf Adapter*), D) cap to block ambient light (*Measurement Chamber v2 Lid*), E) plugs for unused holes (*Measurement Chamber v2 Plug*).

The dimensions of the sample chamber are 88.1 x 78.8 x 44.0 mm (Fig. 4) and consists of 6 parts: an optional base, main chamber, main chamber lid intended for centering a 1.5 mL micro-centrifuge tube, a cap to block ambient light and 2 plugs for unused holes in the main chamber. Due to the size of the parts it is recommended to reduce the print speed to 40 mm/s to achieve higher-quality results. The chamber should be printed with 100 % infill with a layer height of 0.2 mm or less, which would take approx. 11 h 33 min and 93 g (31.17 m) of filament.

To print all the parts necessary it would take 3 prints, 27 h 13 min, and 262 g of filament.

#### 2.4.3 Second iteration

**Fig. 5:**
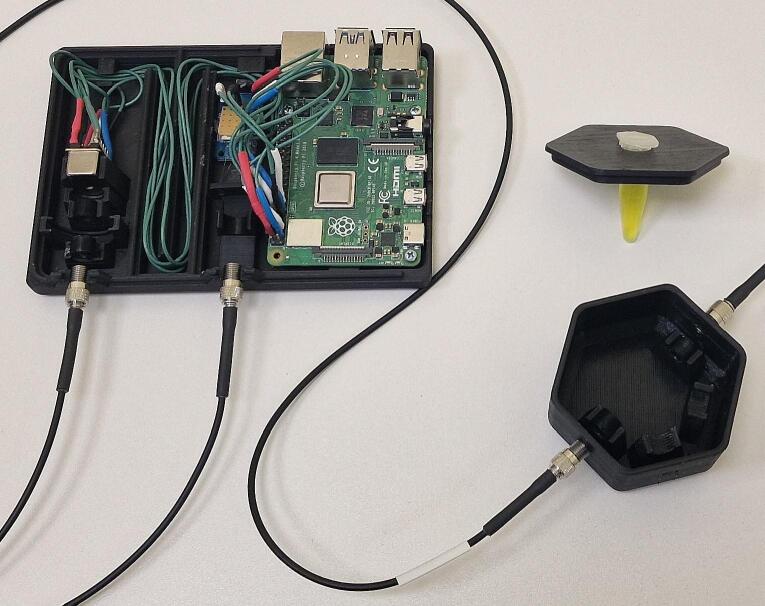
First iteration of the design fully assembled (excluding the case lid).

The dimension of the instrument housing unit is 97.0 x 121.0 x 38.0 mm (Fig. 4) and consists of 4 parts. The base and the lid should be printed with at least 50 % infill with a layer height of 0.2 mm or less, which would take approx. 5 h 11 min and 44 g (14.77 m) of filament to print the base and 7 h 1 min and 61 g (20.32 m). Optical configuration is contained within a lens tube (Fig. 5, E). It is advised to reduce the print of these components with a speed of 50 mm/s to achieve a higher quality final product. The lens tube has to be printed along the z-axis with ironing enabled to achieve the concentricity required by the lens setup. Fabrication of the lens tube will take approx. 32 min and 4 g (1.49 m) of filament. The LED adapter can be printed horizontally and will take approx. 27 min and 4 g (1.32 m) of filament.

#### 2.4.4 Sample chambers used with the second iteration design

**Fig. 6:**
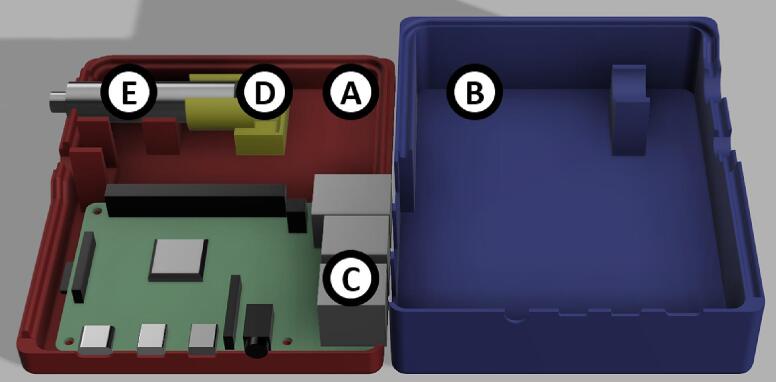
Digital rendering of the whole instrument housing unit. A) Base of the housing unit (*Box v2 Base)*, B) lid of the housing unit (*Box v2 Lid*), C) location of Raspberry Pi 4 model B, D) LED adapter for the lens tube (*Box v2 LED Adapter*), E) lens tube (*LEDTube*).

**Fig. 7:**
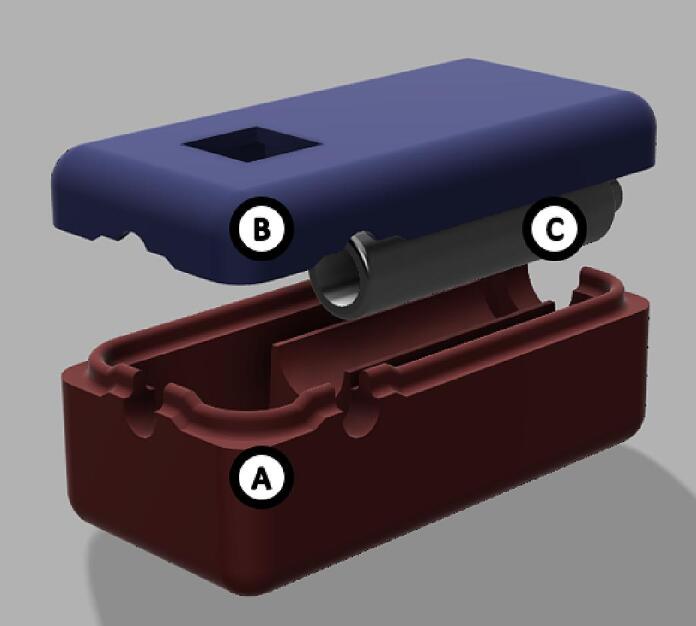
Digital rendering of the sample chamber used with the second iteration design. A) the base of the sample chamber, B) the lid of the sample chamber, and C lens tube.

**Fig. 8:**
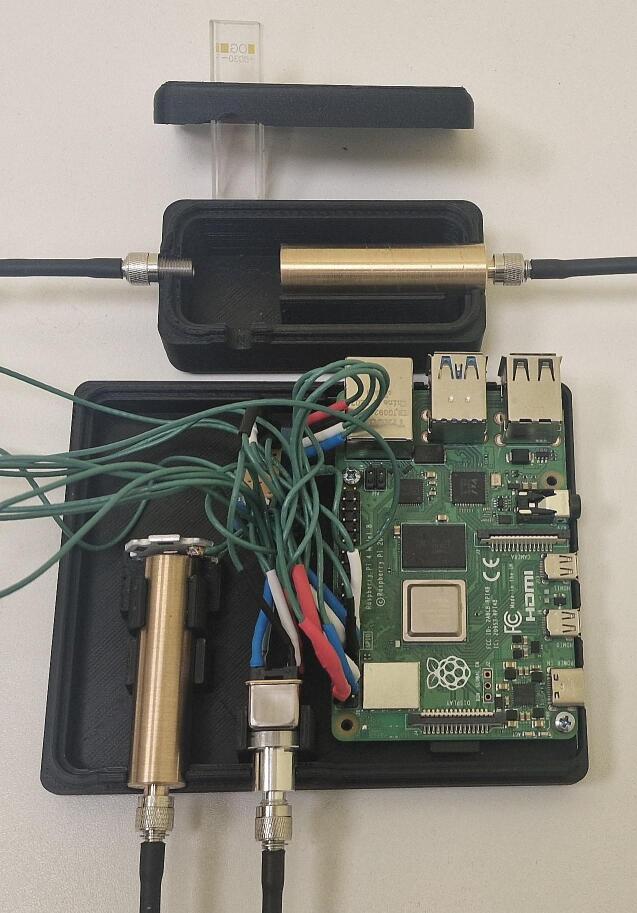
Second iteration of the design fully assembled (excluding case lid).

The dimensions of the sample chamber are 33.3 x 38.5 x 78.5 mm (Fig. 6) and it consists of 3 parts: a base, a lid, and another lens tube. The chamber and the lid (optionally the lens tube) should be printed with 100 % infill with a layer height of 0.2 mm or less, which would take approx. 9 h 21 min and 71 g (23.95 m) of filament. (See Fig. 7, Fig. 8).

To print all the parts necessary it would take 3 prints, 22 h 10 min, and 184 g of filament.

### 2.5 Design files summary

Design files for the first iteration design:

**Table:** 

**Design file name**	**File type**	**Open source license**	**Location of the file**
BoxBase	.stl file	GNU General Public License v3.0	*https://doi.org/10.17605/OSF.IO/938QV*
BoxLid	.stl file	GNU General Public License v3.0	*https://doi.org/10.17605/OSF.IO/938QV*
BoxLEDHolder	.stl file	GNU General Public License v3.0	*https://doi.org/10.17605/OSF.IO/938QV*
BoxLEDLense	.stl file	GNU General Public License v3.0	*https://doi.org/10.17605/OSF.IO/938QV*
Measurement Chamber v2 Base	.stl file	GNU General Public License v3.0	*https://doi.org/10.17605/OSF.IO/938QV*
Measurement Chamber v2 Crystal Holder	.stl file	GNU General Public License v3.0	*https://doi.org/10.17605/OSF.IO/938QV*
Measurement Chamber v2 Epindorf Addapter	.stl file	GNU General Public License v3.0	*https://doi.org/10.17605/OSF.IO/938QV*
Measurement Chamber v2 Lid	.stl file	GNU General Public License v3.0	*https://doi.org/10.17605/OSF.IO/938QV*
Measurement Chamber v2 Plug	.stl file	GNU General Public License v3.0	*https://doi.org/10.17605/OSF.IO/938QV*
Measurement Chamber v2	.stl file	GNU General Public License v3.0	*https://doi.org/10.17605/OSF.IO/938QV*

Design files for the second iteration design:

**Table:** 

**Design file name**	**File type**	**Open source license**	**Location of the file**
Box v2 Base	.stl file	GNU General Public License v3.0	*https://doi.org/10.17605/OSF.IO/BCAQW*
Box v2 LED Adapter	.stl file	GNU General Public License v3.0	*https://doi.org/10.17605/OSF.IO/BCAQW*
Box v2 Lid	.stl file	GNU General Public License v3.0	*https://doi.org/10.17605/OSF.IO/BCAQW*
LEDTube	.stl file	GNU General Public License v3.0	*https://doi.org/10.17605/OSF.IO/BCAQW*
Measurement Chamber v3 Base	.stl file	GNU General Public License v3.0	*https://doi.org/10.17605/OSF.IO/BCAQW*
Measurement Chamber v3 Lid	.stl file	GNU General Public License v3.0	*https://doi.org/10.17605/OSF.IO/BCAQW*
LEDTubeSTEP	.step file	GNU General Public License v3.0	*https://doi.org/10.17605/OSF.IO/BCAQW*

## 3 Bill of materials summary

**Table:** 

**Designator (**Fig. 1**)**	**Component**	**Number**	**Cost per unit − currency**	**Total cost − currency**	**Source of materials**	**Material type**
11	Raspberry Pi 4b 2 GB	1	57 €	57 €	Raspberry Pi	Electronics/semiconductors
8	C12880MA spectrometer	1	170 €	170 €	Hamamatsu	Electronics/semiconductors
9	ADS1115 A/D converter	1	8 €	8 €	TME	Electronics/semiconductors
10	YJD20N06A MOSFET	1	0.17 €	0.17 €	TME	Electronics/semiconductors
5	370 nm LED	1	2.55 €	2.55 €	TME	Electronics/semiconductors
5	480 nm LED	1	0.25 €	0.25 €	Alibaba	Electronics/semiconductors
10	Resistor 2.2 Ω	1	0.01 €	0.01 €	Local hardware store	Electronics/semiconductors
10	Resistor 6.8 Ω	1	0.01 €	0.01 €	Local hardware store	Electronics/semiconductors
N/A	PETG filament for 3D printing case base, cover and the external measurement chamber	262 g	0.016 €	4.20	Gembird	3D printing/polymer
4	SMA screws	4	11 €	44	Thorlabs	Optics/Inorganic
4	Optical fiber	2	22 €	44	Syronoptics	Optics/Inorganic
6	Generic laser collimating lens M9*0.5	2	3.5 €	7	Alibaba	Optics/Inorganic

## 4 Build instructions

It is recommended that the software part of the system setup is completed first to easier isolate potential hardware setup issues and to make these issues easier to diagnose. To reproduce the complete system setup used within this article, you would need to start with a full setup of Raspberry Pi with an operating system (OS). The instructions of how to do that can be found on the official Raspberry Pi webpage under their documentation section [20]. For the purposes of this project, during the OS installation phase in the *Raspberry Pi Imager*, the “Raspberry Pi OS Full (64-bit)” option was chosen when installing the software to a microSD card. Note that not all Raspbian versions are compatible with all Raspberry Pi versions, so double check what model Pi you are using and which OS choices support it (i.e. 64-bit OS can only be installed on Raspberry Pi 3, 4, 400, and 5).

After you have successfully prepared your microSD card and inserted it into the Raspberry Pi, make sure to first connect your monitor to the HDMI port before connecting the Pi to power, as HDMI won't output if you do it in the opposite order.

When you finish the final steps of the OS setup and the system boots to the desktop, some communication interfaces will need to be enabled. At the top left of the screen, there will be an application menu. Go to *Preferences −> Raspberry Pi Configuration −> Interfaces* and enable the *I2C* interface. Here, you can also enable other interfaces like *SSH* and *VNC* for remote control (instructions not included here) and other communication protocols of interest if the system is planned to be expanded upon in the future. If an OS version with no desktop environment was installed, use the terminal command “*sudo raspi-config*”, then navigate the menu into the interfaces and enable what you need. Reboot the device when prompted.

An internet connection to the Raspberry Pi will be required for the following steps. To ensure the system is up to date, open the terminal and run the following commands:

sudo apt

updatesudo apt upgrade

If you chose an OS with no Python or pip (Python Package Index) preinstalled, run:

sudo apt install python3 python3-pip

Next, the library used to communicate with the ADS1115 chip has to be installed. There are various open source Python libraries for this specific chip, but the one used within this article is made by Adafruit [21] and can be installed for the current user by this command in the terminal:

pip3 install adafruit-circuitpython-ads1x15

The argument “--break-system-packages'' has to be appended to this command to install this package starting from Debian 12 due to Python Enhancement Proposal 668 (PEP668) [22]. Alternatively, a virtual environment can be used instead as suggested by PEP668. To finalize the setup, the software provided in this article to run the microspectrometer system can be downloaded at [23] under the *Hamamatsu C12880MA Python library for Raspberry Pi* directory and can be run from anywhere in the system as long as the *C12880MA* control library is properly imported. For simplicity, it is advised to leave the files in the same directory. The package includes three files − C12800MA control library (*libC12880MA.py*), a simple usage example printing out spectra (*example.py*), and a slightly more advanced example with a graphical user interface (GUI) (*example_GUI.py*). The file list can be found in Table 1.

**Table 1:** List of the provided Python files.

**File name**	**File type**	**Open source license**	**Location of the file**
libC12880MA	.py file	GNU General Public License v3.0	*https://doi.org/10.17605/OSF.IO/WY852*
example	.py file	GNU General Public License v3.0	*https://doi.org/10.17605/OSF.IO/WY852*
example_GUI	.py file	GNU General Public License v3.0	*https://doi.org/10.17605/OSF.IO/WY852*

To run the example using the GUI, an additional dependency has to be installed from the terminal:

sudo apt install python3-pyqtgraph

For now, power down the system.

**Fig.9:**
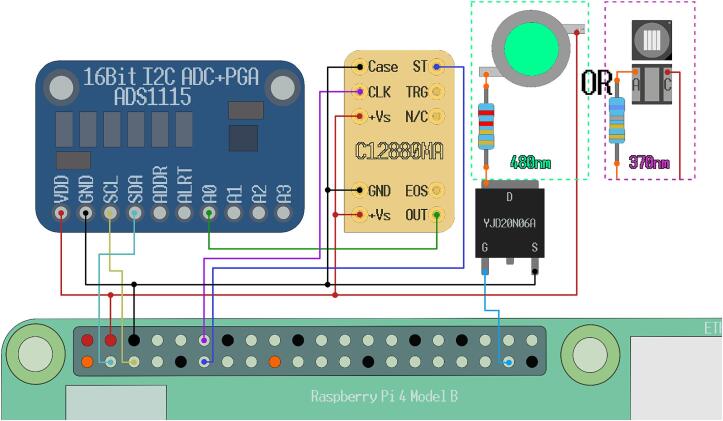
Schematic drawing of the system and its wiring configuration.

After the software has been set up, all the hardware components have to be wired up to each other. The necessary wiring configuration is shown in Fig. 9. Note the distances between the individual component locations within the 3D design models to cut off proper wire lengths. It is highly recommended that soldered connections are made for stability inside the case instead of using pin headers with solderless wires, although a rigid 14-pin (4 pins wide, 7 long) dip is recommended for the C12880MA to avoid damaging the sensor during soldering due to overheating, instead solder wires onto the 14-pin dip and carefully plugging the spectrometer inside it without bending its pins.

The components shown within the dotted square in Fig. 9 are interchangeable—LEDs with their corresponding resistors. If a different LED than the one listed here is used, the resistor necessary to limit the power delivery to it has to be calculated and added to the system. Let us look at the 480 nm LED calculation as an example. The resistance (*R*) necessary is calculated based on the current draw of the LED (*I_f_ = 0.6 A*), its operating forward voltage (*V_f_ = 3.6 V),* and the output of the Raspberry Pi power supply (*V = 5 V):*

R = (V − V_f_)/I_f_ = 2.33 Ω.

The necessary power (*P*) rating of the resistor can be calculated by

P = RI^2^ = 0.84 W,

therefore a resistor with at least 1 W power rating has to be used.

Within this example, 480 nm + 2.2 Ohm and 370 nm + 6.8 Ohm pairs for LED + resistor will be used. Additionally, it is recommended that these LEDs are installed on cooling pads, as the power output can reach over a Watt of power.

It would be wise to connect each part of the system individually on a breadboard first and make sure it works properly before soldering everything together.

## 5 Operation instructions

After the build is complete, boot up the device and open the previously downloaded directory containing the Python files [23]. To begin measurement, simply run the *example.py* script. The resulting spectra and some related data of interest will be continuously printed out in the terminal. Spectral analysis, data storage, plotting, etc. can be performed depending on the needs of the end-user by modifying the code. The Python documentation [24] includes various examples of how to program these interactions − simply use the search function − or perform general online search engine queries. Comments are included next to snippets of code to explain what a specific part of the code does to make it easier to modify.

If only measurement acquisition is necessary, there are 3 parameters of interest you would want to modify − exposure time, number of accumulations, and the duty percent value of LED PWM. A longer exposition time will naturally lead to a higher amount of light collected. Increasing the number of accumulations will make the software repeatedly take measurements at the same exposure and sum up the intensity data at each corresponding wavelength, leading to smoother spectra by improving the signal-to-noise ratio. Generally, regulating the LED PWM duty percent value is necessary to find a balance between inducing fluorescence and the resulting excitation intensity in case the sensor reaches its limits and is underexposed or overexposed.

**Fig.10:**
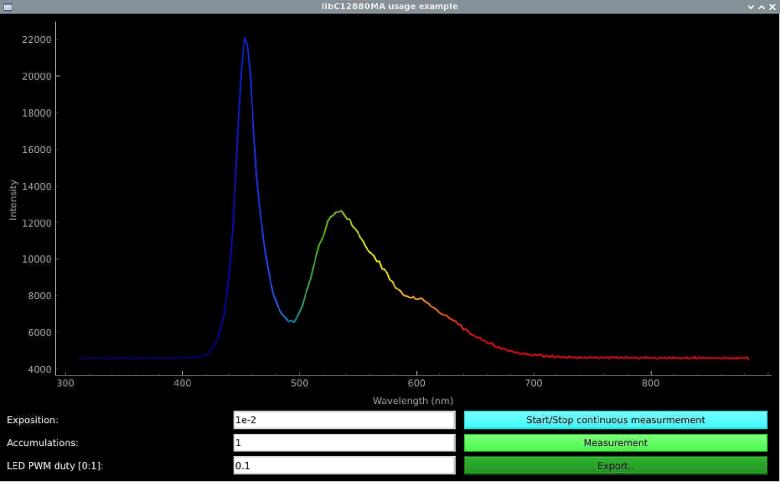
Screenshot of the *example_GUI.py* generated graphical user interface during measurement of a computer screen spectra.

To ease access for a wider audience to reproduce this spectroscopic system, an extra file named *example_GUI.py* is included in the package, which includes a more advanced example of data output from the spectrometer, allowing single-shot or real-time graph plotting, spectrometer input parameter control, and data exporting, all within a simple GUI (Fig. 10). Step-by-step instructions on how to acquire a spectrum with each of the example programs and additional usage notes are included in the linked OSF repository under the Wiki section of the project [23].

## 6 Validation and characterization

### 6.1 Spectral calibration

The paper outlines a spectral correction process consisting of two steps: correcting the intensity over the entire spectrum and correcting the wavelength.

**Fig. 11:**
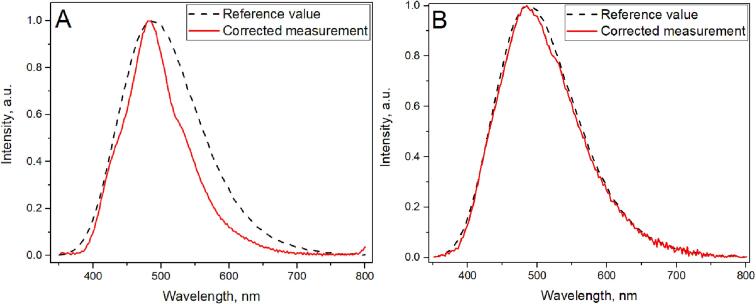
Photoluminescence Measurements of CdWO_4_ with Hamamatsu C12880MA (red line) and FLS1000 (reference, dashed line): (A) Raw Measurements, (B) Spectrally Corrected.

**Fig. 12:**
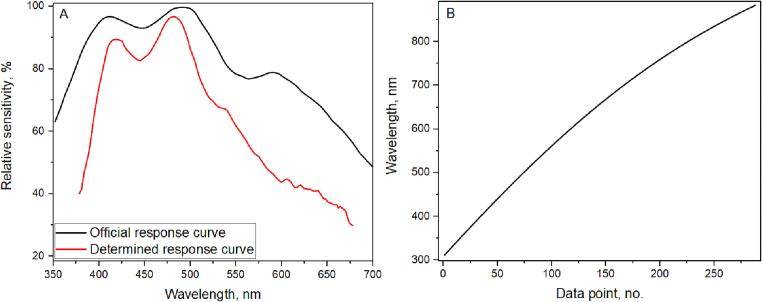
The official and the determined spectral sensitivity of the used Hamamatsu C12880MA unit **(A)** and the relation between data points and wavelength of our specific unit, included in the package **(B)**.

Hamamatsu offers spectral response data in the datasheet, providing relative sensitivity over the spectral range. Wavelength correction (spectral linearity, Fig. 12. B) is provided for each spectrometer coming out of production. We use the factory-provided wavelength correction across the spectrum, but the spectral sensitivity curve needs calibration as it depends on the final optical system. To obtain the spectral correction curve, we measured the CdWO_4_ spectrum in our system and a high-quality spectrometer-Edinburgh Instruments FLS1000, (Fig.11.A). Using these spectra, we obtained the sensitivity spectral distribution of the system (Fig.12.A), allowing us to correct measurements from the Hamamatsu C12880MA and obtain a reliable CdWO_4_ photoluminescence spectrum. Measurements were conducted using 375 nm excitation, FLS1000 used a Xenon lamp, filtered by a double-monochromator as its source. For measurements with the Hamamatsu C12880MA system, the integrated 370 nm LED was used.

This correction file can be applied effectively to other spectral measurements. Note that this specific correction applies to the 400–700 nm range; for effective correction beyond 700–800 nm, a phosphor with luminescence in this red range must be used.

If you are unable to compare with standards and measure using high-end spectrometers or do not wish to perform the correction, a typical spectral response curve can be found in the Hamamatsu C12880MA specification sheet. It will work fair in most applications. A comparison of both sensitivity curves is shown in Fig. 12. A.

### 6.2 Measurement examples

#### 6.2.1 Luminescence

**Fig. 13:**
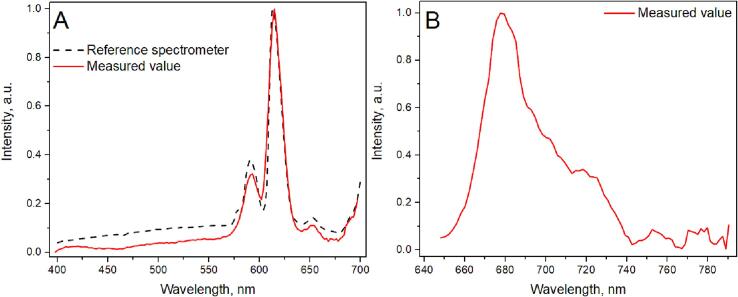
Photoluminescence measurement using built-in UV diode with sensitivity correction for aluminosilicate with europium(III) fluoride with tin oxide **(A)** and fluorescence of chlorophyll *A* solution in ethanol **(B)**. The reference spectrum was acquired using the Andor DV420A-BU2 sensor.

**Fig. 14:**
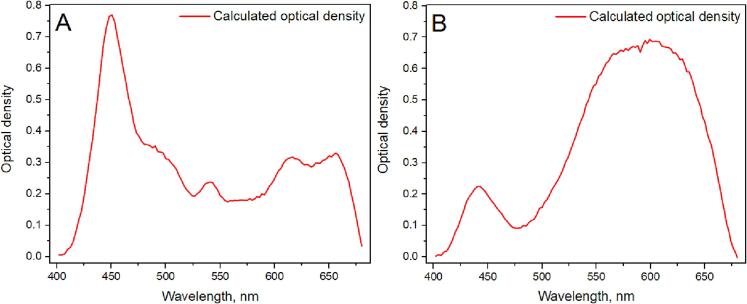
Measured and Calculated optical density of chlorophyll *B* solution in ethanol **(A)** and optical density of ferrocyanide in ethanol solution **(B)**.

Our device is versatile, operating in various configurations such as luminescence, scattering, and absorption. Our testing focused on evaluating its performance in specific luminescence applications. In particular, we tested the system by recording UV diode-excited luminescence of aluminosilicate with europium(III) fluoride and tin oxide (Fig. 13 A). The results demonstrated a remarkable similarity to those obtained using high-end commercial spectroscopy systems. Notably, the spectral resolution approached the limits of the Hamamatsu C12880MA (claimed 10–15 nm by the manufacturer). (see Fig 14).

In an effort to expand our testing beyond inorganic materials, we examined the fluorescence of Chlorophyll *A* solution in ethanol. Despite encountering a weaker luminescence signal accompanied by increased noise, the experiment showcased the feasibility of the device in handling organic material measurements. This broader testing spectrum further reinforces the adaptability and potential of our device across a range of luminescent applications.

#### 6.2.2 Absorption

Absorption measurements were conducted to illustrate the feasibility of performing absorption measurements with the constructed system. 2 readily available substances were chosen: chlorophyll *B* and ferrocyanide. Both biological substances were diluted with pure laboratory-grade ethanol. A cool white LED was used to simulate a full visible light spectrum. The light *I_0_* was measured with an empty cuvette and then with a cuvette filled with one of the aforementioned solutions (*I*). Initially, the transmission coefficient was determined by employing formula (1), after which optical density was calculated with formula (2).

(1) $$T=I/I_{0}$$

(2) $$\mathrm{OD}=-log_{10}\left(T\right)OD$$

The obtained data are directly comparable to measurements conducted by other scientific groups [25] which leads to the assumption that the constructed system is fully suitable for performing absorption measurements.

### 6.3 Performance summary

A highly configurable fiber-based system was developed. This spectroscopy system demonstrates reasonably good performance in a variety of applications and configurations when the cost of the system is considered, compared to the commercial high-end systems. While collecting light in fibers can be a demanding operation, when done properly, it offers flexibility and convenience of usage after the system is fully built, which is especially useful in advanced circumstances and for in-situ research.

### CRediT authorship contribution statement

**Gatis Tunens:** Writing – review & editing, Validation, Software, Resources, Investigation. **Ernests Einbergs:** Writing – review & editing, Visualization, Validation, Resources, Investigation. **Katrina Laganovska:** Methodology, Investigation, Formal analysis. **Aleksejs Zolotarjovs:** Writing – review & editing, Methodology, Conceptualization. **Karlis Vilks:** Validation. **Linards Skuja:** Writing – review & editing, Validation, Methodology, Formal analysis, Data curation. **Krisjanis Smits:** Writing – review & editing, Writing – original draft, Visualization, Supervision, Resources, Project administration, Methodology, Investigation, Funding acquisition, Formal analysis, Data curation, Conceptualization.

## Declaration of competing interest

The authors declare that they have no known competing financial interests or personal relationships that could have appeared to influence the work reported in this paper.
